# Psoas muscle mass index and peak expiratory flow as measures of sarcopenia: relation to outcomes of elderly patients with resectable esophageal cancer

**DOI:** 10.3389/fonc.2023.1303877

**Published:** 2023-11-28

**Authors:** Mingzhi Zhang, Yaqiong Xiong, Mengzhou Chen, Dafu Xu, Keping Xu, Wenze Tian

**Affiliations:** ^1^ Department of Thoracic Surgery, The Affiliated Huaian No.1 People’s Hospital of Nanjing Medical University, Huaian, China; ^2^ Department of Respiratory and Critical Care Medicine, The Affiliated Huaian No.1 People’s Hospital of Nanjing Medical University, Huaian, China

**Keywords:** esophageal cancer, sarcopenia, psoas muscle mass index, peak expiratory flow, complications, survival

## Abstract

**Objectives:**

The objective of this study is to investigate whether the evaluation of postoperative outcomes or overall survival in patients who undergo surgery for esophageal cancer can be achieved by assessing sarcopenia using psoas muscle mass index and peak expiratory flow.

**Methods:**

This retrospective study analyzed the clinical data of 356 elderly patients (≥ 65 years) who had undergone radical surgery for esophageal cancer. Muscle mass and muscle strength were assessed by psoas muscle mass index (bilateral psoas area/height^2^) and peak expiratory flow, using preoperative computed tomography and spirometry, respectively. Sarcopenia is defined as a condition where both the psoas muscle mass index and peak expiratory flow fall below their gender-specific cutoff values. Survival and postoperative complications were compared between patients with and without sarcopenia.

**Results:**

Out of the 356 elderly individuals diagnosed with esophageal cancer, 84 patients (23.6%) were found to have sarcopenia. The group with sarcopenia showed a notably higher occurrence of postoperative pneumonia (29.8% vs 16.9%, P < 0.001) and anastomotic leak (9.5% vs 3.7%, P < 0.05) compared to those without sarcopenia. Additionally, a multivariate analysis concluded that sarcopenia independently acted as a risk factor for postoperative pneumonia, possessing an odds ratio of 1.90 (P < 0.05). The survival rate after 3 years for individuals with sarcopenia was considerably lower than those without sarcopenia (57.8% vs 70.2%, P < 0.05). Sarcopenia was identified as an unfavorable prognostic factor for overall survival, with a hazard ratio of 1.51 (P < 0.05).

**Conclusions:**

Preoperative sarcopenia diagnosed by psoas muscle mass index and peak expiratory flow is associated with reduced overall survival and adverse postoperative outcomes among elderly individuals suffering from esophageal cancer.

## 1 Introduction

Esophageal cancer (EC) ranks as the sixth leading cause of cancer-related death on a global scale (1). A considerable proportion of individuals diagnosed with esophageal cancer encounter varying degrees of malnutrition, ranging from 60% to 85%, making it the most prevalent malnutrition among all types of malignant tumors (2). The presence of malnutrition plays a significant role in determining the treatment response and prognosis of patients grappling with esophageal cancer. Moreover, this category of patients typically experiences notable alterations in body composition, such as a decrease in lean muscle mass and adipose tissue, which stem from cancer-related malnutrition and the activation of inflammatory cytokines. The severity of these alterations is further intensified by dysphagia resulting from tumor stenosis (3–5).

Sarcopenia is a degenerative disorder of the skeletal muscle, distinguished by a decline in both muscle mass and strength (6, 7). It frequently presents in individuals diagnosed with esophageal cancer, with prevalence rates varying from 16% to 75% (8). Studies have provided evidence that sarcopenia significantly heightens the risk of postoperative complications like pneumonia and anastomotic leak in esophageal cancer surgical patients (9–11). Additionally, it has been noted that sarcopenia negatively impacts overall survival and disease-free survival rates among these patients (9, 12, 13).

As per the consensus of the Asian Working Group on Sarcopenia and the European Working Group on Sarcopenia, the diagnosis of sarcopenia necessitates an assessment from two perspectives - muscle mass and muscle strength. Various methods are available to evaluate muscle mass, such as dual-energy X-ray absorptiometry, bioelectrical impedance analysis, magnetic resonance imaging, and computed tomography (CT). In addition, diminished muscle strength has been acknowledged as a vital diagnostic feature for sarcopenia and a reliable predictor of unfavorable consequences.Tests to measure muscle strength encompass grip strength, peak expiratory flow (respiratory muscle strength), and the chair stand test (leg muscle strength) (6, 7, 14).

However, previous investigations into sarcopenia in esophageal cancer have primarily focused on the diagnosis of reduced muscle mass, disregarding the evaluation of muscular strength (11, 15–17). This disregard could be attributed to the laborious process of gauging muscle strength and the accompanying expenses. CT imaging has the capability to assess muscle mass through the utilization of the skeletal muscle index (SMI) and psoas muscle mass index (PMI) (6). In comparison to SMI, PMI offers advantages such as well-defined boundaries, a smaller area of measurement, and the ability to measure individual muscles, rendering it more suitable for the assessment of preoperative patients in clinical practice (18). Peak expiratory flow (PEF) denotes the maximum airflow produced during forced exhalation and exhibits an intrinsic correlation with respiratory muscle strength (19). The study conducted by Ridwan additionally identified that diminished PEF is independently linked to the occurrence of sarcopenia (20). Furthermore, researchers have identified respiratory sarcopenia, characterized by PEF (21, 22). Preoperative CT and spirometry represent standard procedures employed in the staging and evaluation of esophageal cancer patients before surgical intervention. In our study, we employ CT images to capture PMI alterations, allowing for the evaluation of changes in muscle mass, while simultaneously utilizing PEF measurements from spirometry to assess variations in muscle strength. This approach offers a more pragmatic and convenient means of diagnosis.

This retrospective study utilizes the parameters of PMI and PEF to diagnose preoperative sarcopenia in elderly patients diagnosed with esophageal cancer, and investigates the impact of sarcopenia on postoperative complications and overall survival.

## 2 Materials and methods

### 2.1 Study design and participants

This retrospective investigation was carried out at the Department of Thoracic Surgery, located in the Affiliated Huaian No.1 People’s Hospital of Nanjing Medical University. The study enrolled individuals diagnosed with esophageal cancer who underwent surgical intervention from January to December 2020. The investigation focused on patients who fulfilled the following inclusion criteria: (1) age of 65 years or above, (2) received an abdominal CT scan within a fortnight prior to the procedure, (3) pathology reports confirmed the presence of squamous cell carcinoma or adenocarcinoma, and (4) underwent McKeown surgery. Subjects meeting any of the subsequent exclusion criteria were excluded from the study: (1) received neoadjuvant treatment before the operation, (2) lacked comprehensive clinical or imaging data, (3) had other malignancies, (4) necessitated conversion to open surgery during the procedure, or (5) exhibited distant metastasis. This investigation strictly adhered to the ethical principles outlined in the Declaration of Helsinki and received approval from the Medical Ethics Committee of our hospital under the reference number KY-2022-037-01.

### 2.2 Information on clinical parameters

The extracted clinical data consisted of the subsequent details: age, gender, hypertension, diabetes mellitus, smoking history (smoking index ≥400), preoperative pulmonary function test (forced expiratory volume in 1 second [FEV1], peak expiratory flow [PEF]), preoperative laboratory parameters (albumin, pre-albumin), tumor stage (in accordance with the 8th edition of the UICC/AJCC staging criteria for esophageal cancer in 2017), body mass index (BMI), prognostic nutritional index (PNI), short-term outcomes (postoperative complications, hospital length of stay [LOS], 30-day mortality).

The deadline for follow-up was July 31, 2023, and patients were monitored through either outpatient appointments or telephone interviews. Overall survival was defined as the duration from surgery to the last follow-up appointment or death resulting from any cause.

### 2.3 Postoperative complications

Within a 30-day period following esophagectomy, we assessed the occurrence of postoperative complications demanding medical or surgical intervention for management. The diagnostic criteria for postoperative pneumonia detection within a 30-day period after surgery must adhere to the following three conditions concurrently: (1) the presence of at least two chest radiographs and, furthermore, a minimum of one pneumonia diagnosis; (2) fulfillment of at least one of the subsequent requirements: age ≥70 years, peripheral blood WBC count 12 × 10^9^/L, and fever accompanied by altered consciousness (body temperature >38°C); (3) the manifestation of a minimum of two of the subsequent indications: occurrence of purulent sputum or alteration in sputum characteristics, heightened respiratory secretions, necessity to increase the frequency of sputum generation, or presence of dyspnea.

The identification of an anastomotic leak was achieved by means of esophagography or fistulography, in addition to the consequent discharge of saliva through the fistula. Pleural effusion and arrhythmia were diagnosed based on the criteria established by the Esophagectomy Complications Consensus Group (23). Overall complications are defined as the occurrence of one or more of the above.

### 2.4 Determination of sarcopenia

Retrospective collection and analysis of abdominal CT images from patients diagnosed with esophageal cancer took place prior to surgery, within a period of 14 days. Measurement of muscles was conducted within a HU range of −29 to +150 HU, and adjustments were made manually to address any issues pertaining to tissue boundaries. The calculation of PMI (bilateral psoas area/height^2^) involved adding the left and right psoas areas of the L3 segment and normalizing them in relation to height.

Hamaguchi et al. developed a new set of diagnostic criteria to evaluate low muscle mass on CT scans in the Asian population. Accordingly, the PMI thresholds for sarcopenia in men and women were 6.36 cm^2^/m^2^ and 3.92 cm^2^/m^2^, respectively (24). A Japanese cross-sectional study including 681 community-dwelling elderly individuals determined sex-specific thresholds for PEF to be 4.40 L/s for males and 3.21 L/s for females (21). Preoperative sarcopenia diagnosis relied on the presence of PMI and PEF measurements falling below the gender-specific cutoff values.

### 2.5 Statistical analysis

The normality of the collected data was evaluated by visually examining histograms and conducting the Shapiro-Wilk’s W-test. If the data followed a normal distribution, it was presented as the mean and standard deviation (SD); otherwise, it was presented as the median and interquartile range (IQR). Categorical data was presented as the number and percentage. In order to examine distinctions among groups regarding continuous data, we employed the Mann-Whitney U test, whereas the Pearson chi-squared test or Fisher’s exact probability method was utilized for categorical data. The determination of cutoff values for continuous variables was based on the median of the cohort (age [70 years]), defined class (BMI [18.5 kg/m^2^, 24 kg/m^2^]), predicted FEV1% [80%]), and thresholds reported in previous studies (pre-albumin [160 mg/L]) (25, 26).

Survival curves were generated using Kaplan-Meier analysis, and the log-rank test was utilized to compare differences. We developed a logistic regression model with the intent of identifying preoperative prognostic factors specifically related to postoperative pneumonia. The model incorporated all variables found to have a significance level of P < 0.1 in the initial univariate analysis. Univariate and multivariate analyses were conducted using Cox proportional hazards models, and variables with P values less than 0.1 at the univariate level were included in the multivariate model. Hazard ratios (HR) and corresponding 95% confidence intervals (CI) were calculated to determine the strength of associations. To conduct statistical analyses, we employed two software programs: SPSS version 26.0 developed by IBM Corp in Armonk, NY, USA and Prism 8 designed by GraphPad Software in La Jolla, CA, USA.

## 3 Results

During the period from January 2020 to December 2020, a total of 428 older individuals (aged ≥65 years) diagnosed with esophageal cancer underwent the McKeown surgical procedure at our medical facility. None of these patients were subjected to neoadjuvant therapy. Out of these individuals, 21 did not have CT scans performed within a 2-week window prior to the operation, 15 patients lacked complete clinical data, and 36 patients were lost to follow-up. Subsequently, we included a total of 356 patients who fulfilled the predetermined inclusion criteria for our study. Among the study population, there were 235 male patients and 121 female patients, with a median age of 70 years.

### 3.1 Characteristics between sarcopenic and non-sarcopenic patients

According to our criteria, a total of 84 patients (23.6%) were diagnosed with preoperative sarcopenia, while the remaining 272 patients did not exhibit signs of sarcopenia. **Table 1** contains the clinical characteristics of both the sarcopenic and non-sarcopenic patients. It was observed that the sarcopenic patients displayed a significantly higher age compared to the non-sarcopenic patients. The occurrence rates of sarcopenia among the three age groups, specifically individuals aged 65-70, 71-75, and over 75, were found to be 15.7%, 30.7%, and 38.6% respectively. Additionally, when considering sarcopenia as a grouping factor, notable disparities were noted in terms of BMI (P < 0.001), FEV1% (P < 0.001), and pre-albumin levels (P = 0.006) within the various groups. Furthermore, it was observed that sarcopenic patients experienced a lengthier duration of surgical procedures compared to their non-sarcopenic counterparts (P = 0.048). Nevertheless, no significant distinctions were identified concerning other factors related to the host, laboratory indicators, or tumor-related factors between these two groups.

**Table 1:** Characteristics of patients with or without sarcopenia.

Variables	Total (n = 356)	Sarcopenia	Non-sarcopenia	*P* value
		(n = 84, 23.6%)	(n = 272, 76.4%)	
Gender, n (%)				0.683
Male	235 (66.0)	57 (67.9)	178 (65.4)	
Female	121 (34.0)	27 (32.1)	94 (34.6)	
Age (y), Median (IQR)	70 (67–74)	72 (68-76)	68.5 (67-72)	<0.001
Hypertension, n (%)				0.853
No	254 (71.3)	59 (70.2)	195 (71.7)	
Yes	102 (28.7)	25 (29.8)	77 (28.3)	
Diabetes mellitus, n (%)				0.379
No	317 (89.0)	77 (91.7)	240 (88.2)	
Yes	39 (11.0)	7 (8.3)	32 (11.8)	
Smoking status, n (%)				0.383
No	259 (72.8)	58 (69.0)	201 (73.9)	
Yes	97 (27.2)	26 (31.0)	71 (26.1)	
BMI (kg/m^2^), n (%)				<0.001
Underweight	22 (6.2)	4 (4.8)	18 (6.6)	
Normal weight	213 (59.8)	65 (77.4)	148 (54.4)	
Over weight	121 (34.0)	15 (17.8)	106 (39.0)	
Location, n (%)				0.776
Upper	44 (12.4)	9 (10.7)	35 (12.9)	
Middle	252 (70.8)	62 (73.8)	190 (69.8)	
Lower	60 (16.8)	13 (15.5)	47 (17.3)	
pT stage, n (%)				0.105
1	83 (23.3)	14 (16.7)	69 (25.4)	
2	79 (22.2)	16 (19.0)	63 (23.2)	
3	194 (54.5)	54 (64.3)	140 (51.5)	
pN stage, n (%)				0.904
0	229 (64.3)	57 (67.9)	172 (63.2)	
1	82 (23.0)	17 (20.2)	65 (23.9)	
2	32 (9.0)	7 (8.3)	25 (9.2)	
3	13 (3.7)	3 (3.6)	10 (3.7)	
FEV1%, Mean ± SD	90.4 ± 19.9	80.3 ± 19.1	93.5 ± 19.2	<0.001
PNI, Mean ± SD	51.3 ± 4.9	51.0 ± 4.7	51.4 ± 5.0	0.706
Albumin (g/L), Mean ± SD	43.7 ± 4.1	43.4 ± 3.6	43.7 ± 4.3	0.534
Pre-albumin (mg/L), Mean ± SD	195.2 ± 33.7	186.3 ± 34.4	197.9 ± 33.0	0.006
Operation time (h), Mean ± SD	3.8 ± 1.0	4.0 ± 1.0	3.8 ± 1.1	0.048
PMI (cm^2^/m^2^), Mean ± SD				
Male	5.5 ± 1.4	4.4 ± 1.1	5.8 ± 1.3	
Female	4.0 ± 1.1	2.9 ± 1.2	4.4 ± 1.1	
PEF (L/s), Mean ± SD				
Male	5.5 ± 1.6	3.6 ± 0.6	6.1 ± 1.4	
Female	4.0 ± 1.0	2.8 ± 0.4	4.4 ± 0.9	

IQR, interquartile range; BMI, body mass index; FEV1, forced expiratory volume in 1 s; SD, standard deviation; PNI, prognostic nutritional index; PMI, psoas muscle mass index; PEF, peak expiratory flow.

### 3.2 Sarcopenia and postoperative complications

The occurrence of postoperative pneumonia exhibited a notably higher prevalence in the group with sarcopenia opposed to the non-sarcopenic group (29.8% vs 16.9%, P < 0.001). Additionally, patients with sarcopenia also manifested elevated rates of anastomotic leak (9.5% vs 3.7%, P = 0.033) and encountered a greater overall incidence of complications when compared to the non-sarcopenic group (**Table 2**). **Table 3** lays out the risk factors for postoperative pneumonia. Through univariate analysis, it was disclosed that age (≥70y), history of smoking, FEV1% (<80%), operation time (≥4h), and sarcopenia were linked to the emergence of postoperative pneumonia. Following adjustment for age (≥70y) and smoking history in the multivariate analysis, sarcopenia was determined as an autonomous risk factor for pneumonia that arises as a consequence of esophageal cancer surgery (OR, 1.902; 95% CI, 1.040-3.479; P = 0.037).

**Table 2:** Postoperative complications with or without sarcopenia.

Variables	Sarcopenia n = 84	Non-sarcopenia n = 272	*P* value
Hospital LOS (d), Mean ± SD	18.3 ± 8.8	16.6 ± 7.3	0.249
Postoperative pneumonia, n (%)	27 (29.8)	44 (16.9)	<0.001
Anastomotic leak, n (%)	8 (9.5)	10 (3.7)	0.033
Pleural effusion, n (%)	10 (11.9)	16 (5.9)	0.064
Arrhythmia, n (%)	19 (22.6)	42 (15.4)	0.127
30-day mortality, n (%)	1 (1.2)	1(0.3)	0.416
Overall complications, n (%)	43 (51.2)	78 (28.7)	<0.001

LOS, length of stay; SD, standard deviation.

**Table 3:** Logistic regression model on postoperative pneumonia using univariate and multivariate analysis.

Variables	Univariate Analysis		Multivariate Analysis	
	Odds Ratio (95% CI)	*P* value	Odds Ratio (95% CI)	*P* value
Gender(male)	0.777 (0.442-1.367)	0.381		
Age (≥70y)	2.086 (1.211-3.593)	0.008	1.772 (1.007-3.117)	0.047
Hypertension	0.971 (0.545-1.729)	0.920		
Diabetes mellitus	0.705 (0.283-1.754)	0.452		
Smoking status	2.203 (1.276-3.804)	0.005	2.176 (1.231-3.845)	0.007
BMI (<18.5, ≥ 24) (kg/m^2^)	1.114 (0.657-1.887)	0.689		
FEV1% (<80%)	1.718 (0.994-2.969)	0.052	1.467 (0.807-2.667)	0.209
pT stage (T_3_)	0.827 (0.491-1.391)	0.474		
pN stage (N_+_)	1.418 (0.834-2.411)	0.197		
Pre-albumin (<160mg/L)	0.851 (0.378-1.918)	0.697		
Operation time(≥4h)	1.750 (1.037-2.954)	0.036	1.386 (0.800-2.400)	0.244
Sarcopenia	2.455 (1.402-4.298)	0.002	1.902 (1.040-3.479)	0.037

CI, confidence interval; BMI, body mass index; FEV1, forced expiratory volume in 1 s.

### 3.3 Prognostic significance of sarcopenia

**Figure 1:**
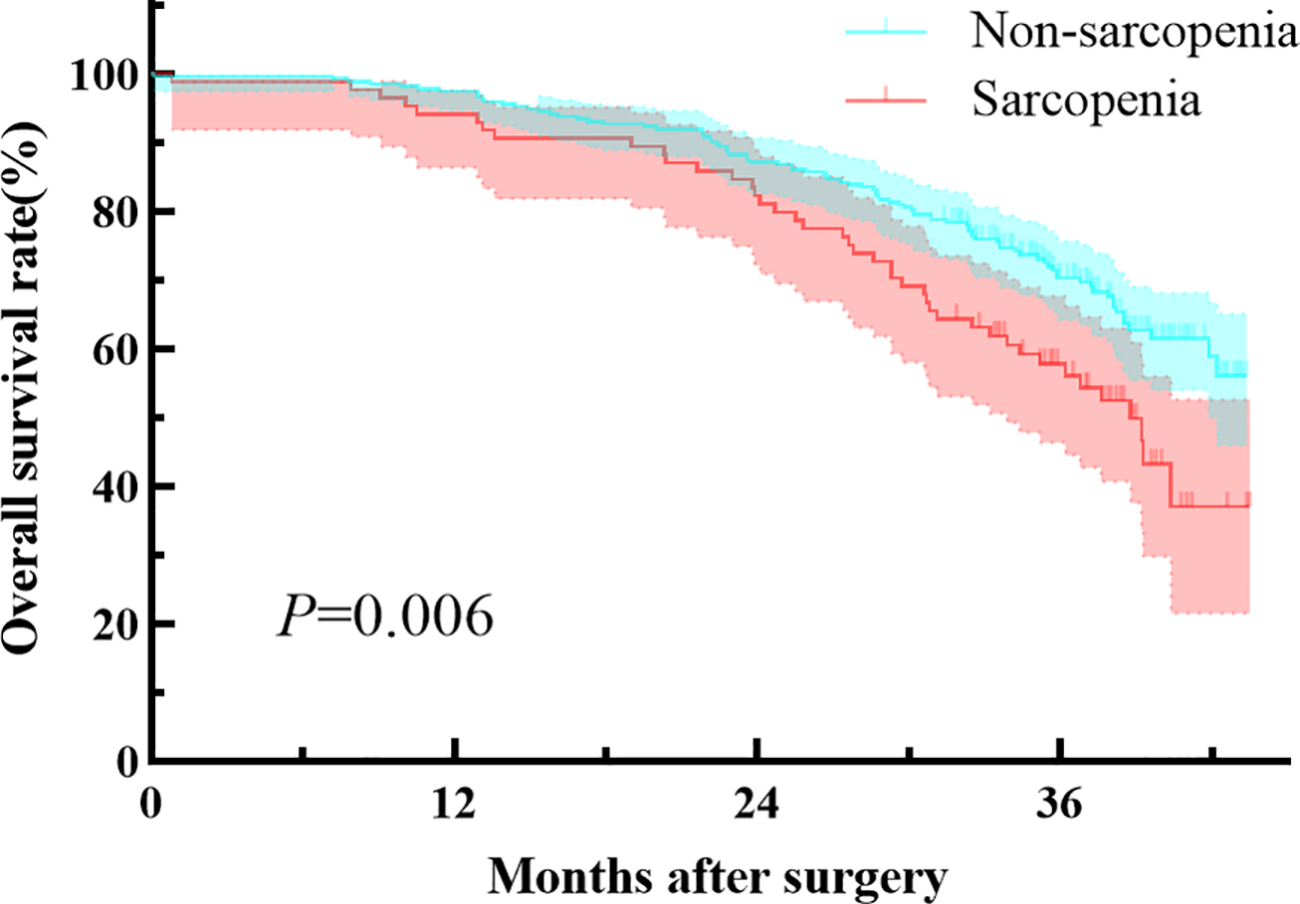
Comparison of survival in patients with or without sarcopenia.

Patients with preoperative sarcopenia had worse overall survival compared to those without sarcopenia, with a median follow-up of 35.1 months (median 34.5 [IQR, 27.6-38.5] vs 35.2 [IQR, 31.9-38.9] months, P=0.006; **Figure 1**). The preoperative sarcopenia group and the non-sarcopenia group showed no statistical difference in the 2-year overall survival rate (82.1% vs 87.1%, P=0.377). However, patients with sarcopenia had significantly lower 3-year survival rate compared to those without sarcopenia (57.8% vs 70.2%, P=0.018). **Table 4** presents the results of the Cox regression analysis of overall survival. The univariate analysis identified age (≥70y), pT stage (T_3_), pN stage (N_+_), and sarcopenia as factors associated with adverse overall survival. Additionally, the multivariate analysis confirmed that the presence of sarcopenia was a significant prognostic factor for overall survival (HR, 1.510; 95% CI, 1.033-2.206; P = 0.033).

**Table 4:** Cox proportional hazard model of clinical characteristics on OS using univariate and multivariate analysis.

Variables	Univariate Analysis		Multivariate Analysis	
	Hazard Ratio (95% CI)	*P* value	Hazard Ratio (95% CI)	*P* value
Gender(male)	0.847 (0.587-1.223)	0.376		
Age (≥70y)	1.713 (1.202-2.440)	0.003	1.534 (1.067-2.205)	0.021
Hypertension	1.080 (0.740-1.575)	0.691		
Diabetes mellitus	0.613 (0.322-1.168)	0.137		
Smoking status	1.323 (0.914-1.915)	0.138		
BMI (<18.5, ≥24) (kg/m^2^)	0.876 (0.616-1.247)	0.463		
FEV1% (<80%)	1.013 (0.695-1.478)	0.946		
pT stage (T_3_)	1.915 (1.326-2.764)	0.001	1.547 (1.057-2.265)	0.025
pN stage (N_+_)	1.959 (1.388-2.764)	<0.001	1.796 (1.257-2.567)	0.001
Pre-albumin (<160mg/L)	1.192 (0.724-1.964)	0.490		
Sarcopenia	1.667 (1.154-2.406)	0.006	1.510 (1.033-2.206)	0.033

CI, confidence interval; BMI, body mass index; FEV1, forced expiratory volume in 1 s.

## 4 Discussion

This study demonstrated that sarcopenia, diagnosed by PMI and PEF, was identified as an independent risk factor for overall survival in elderly patients with resectable esophageal cancer. It was also found to be associated with adverse postoperative outcomes, and independently related to the occurrence of postoperative pneumonia.

Sarcopenia, characterized by progressive loss of muscle mass and strength, is a skeletal muscle disorder. Muscle function can be reliably assessed using muscle strength, which has been demonstrated in recent studies (6). The occurrence of sarcopenia can be indicated by a decrease in muscle strength, which is accompanied by the loss of muscle mass. The diagnosis of sarcopenia can be further supported by confirming the loss of muscle mass (7). The strength of respiratory muscles can be determined by measuring PEF, and low PEF is independently associated with the development of sarcopenia. This association is supported by a recent cross-sectional study conducted in the community (14, 20, 21). Our research findings indicate that patients with low PMI and low PEF have a significantly lower 3-year survival rate. These patients are also more susceptible to complications such as postoperative pneumonia and anastomotic leak. Therefore, the combined assessment of muscle mass and muscle strength provides a comprehensive evaluation of the patient’s skeletal muscle status.

Previous investigations have demonstrated that sarcopenia is linked to inferior overall survival in individuals diagnosed with esophageal cancer post-surgery (27–29). A comprehensive analysis revealed that patients affected by sarcopenia exhibited significantly lower 3-year (51.6% vs. 65.4%, P < 0.001) and 5-year overall survival rates (41.2% vs 52.2%, P = 0.018) in contrast to those without sarcopenia (12). Another systematic review, comprising six studies on long-term outcomes after esophagectomy, arrived at similar findings, underscoring sarcopenia as an autonomous risk factor contributing to unsatisfactory overall survival subsequent to surgery (8). Our research aligns with these outcomes. In patients undergoing neoadjuvant treatment, sarcopenia was also identified as a predictor of poorer overall survival (30–32). Consequently, our study excluded individuals receiving neoadjuvant therapy. Sarcopenia signifies a state of severe malnourishment. Our investigation uncovered that the sarcopenic cohort presented lower pre-albumin levels in comparison to the non-sarcopenic group, potentially leading to detrimental effects on long-term prognosis (33). Moreover, the inferior long-term prognosis can be attributed to the systemic inflammatory response incited by sarcopenia. Skeletal musculature not only functions in exercising and locomotion but also actively engages in intricate immunological and inflammatory processes by secreting diverse cytokines (34–36). Additionally, sarcopenia may heighten the occurrence of adverse reactions to radiotherapy and chemotherapy for esophageal cancer. Individuals affected by sarcopenia demonstrate an augmented susceptibility towards chemotherapy-induced toxic reactions when subjected to platinum-based chemotherapy regimens, which may ultimately result in dosage reduction or even discontinuation of chemotherapy, thereby profoundly influencing treatment outcomes (37–39). Our findings substantially support the clinical value of sarcopenia in nutritional risk assessment and prognostic evaluation.

Various studies have consistently shown that sarcopenia is linked to adverse perioperative results in individuals diagnosed with esophageal cancer, even though the diagnostic approaches employed may differ (40–43). Xu’s study identified sarcopenia, as diagnosed by low PMI, as an independent risk factor for postoperative pneumonia in these patients (44). Fehrenbach’s study also concluded that sarcopenia patients had an increased risk of major complications such as anastomotic leak and postoperative pneumonia, which aligns with our findings (40). However, a meta-analysis indicated that sarcopenia only correlated with postoperative pulmonary complications, not anastomotic leak, cardiac complications, or surgical site infection (8). It’s important to note that differences in diagnostic methods and the age distribution may have influenced these conclusions. Nakashima et al. reported that sarcopenia was an independent risk factor for anastomotic leak in patients aged 65 years and older, but not in those younger than 65 (11). Our study specifically focused on patients aged 65 years and older and found a correlation between sarcopenia and both postoperative pneumonia and anastomotic leak. Age plays a critical role in the development of sarcopenia, as elderly patients often experience more significant malnutrition and have impaired healing abilities, increasing their vulnerability to anastomotic leak (45, 46). Therefore, subgrouping by age is essential in studying sarcopenia. Additionally, elderly patients with sarcopenia are more susceptible to postoperative pneumonia, likely due to a decline in respiratory muscle strength (47). The PEF value reflects patients’ coughing ability, and effective coughing is crucial in preventing postoperative pneumonia (48). Consequently, preoperative detection of sarcopenia can significantly contribute to the perioperative assessment of patients.

A notable strength of this research is its employment of a convenient diagnostic technique that can be derived from CT scans and spirometry conducted before surgery. These procedures do not require extra radiation exposure or intricate measurements in a clinical environment. Furthermore, the study included a substantial number of participants who had undergone surgical removal of esophageal cancer, ensuring a homogeneous cohort. Moreover, there were adequate occurrences of events for each variable in all statistical analyses. The data collection regarding preoperative information and complications was carried out retrospectively in a logical manner, and the outcomes were adjusted for numerous common baseline factors.

There are some limitations to this investigation. Initially, the research was carried out retrospectively in a solitary establishment. Consequently, these discoveries necessitate validation through expansive, population-oriented prospective investigations. Furthermore, the inquiry depended on information concerning muscle mass and muscle strength acquired from spirometry and CT imaging prior to surgery, all at a solitary instance. In order to delve deeper into the prognostic importance of muscle mass and muscle strength in aged patients with esophageal cancer, forthcoming research should encompass longitudinal evaluations of alterations occurring both before and after surgery.

## Data availability statement

The original contributions presented in the study are included in the article/**Supplementary Material**. Further inquiries can be directed to the corresponding authors.

## Author contributions

MZ: Writing – original draft, Formal analysis. YX: Writing – original draft, Formal analysis. MC: Writing – review & editing, Data curation. DX: Writing – review & editing, Data curation. KX: Writing – review & editing. WT: Writing – review & editing.
